# Effect of virtual running with exercise on functionality in pre-frail and frail elderly people: randomized clinical trial

**DOI:** 10.1007/s40520-023-02414-x

**Published:** 2023-05-15

**Authors:** Sara Mollà-Casanova, Elena Muñoz-Gómez, Núria Sempere-Rubio, Marta Inglés, Marta Aguilar-Rodríguez, Álvaro Page, Juan López-Pascual, Pilar Serra-Añó

**Affiliations:** 1 UBIC Research Group, Department of Physiotherapy, Faculty of Physiotherapy, University of Valencia, Gascó Oliag, 5, Valencia, Spain; 2 Instituto Universitario de Ingeniería Mecánica y Biomecánica, Universitat Politècnica de València, Camino de Vera s/n, 46022 Valencia, Spain; 3 Instituto de Biomecánica de Valencia, Universitat Politècnica de València, Camino de Vera s/n, 46022 Valencia, Spain

**Keywords:** Bodily illusion therapy, Frailty, Functionality, Pain, Tone

## Abstract

**Background:**

Virtual mirror therapies could increase the results of exercise, since the mirror neuron system produces an activation of motor execution cortical areas by observing actions performed by others. In this way, pre-frail and frail people could use this system to reach an exercise capacity threshold and obtain health benefits.

**Aim:**

The aim of this study is to evaluate the effects of a virtual running (VR) treatment combined with specific physical gait exercise (PE) compared to placebo VR treatment combined with PE on functionality, pain, and muscular tone in pre-frail and frail older persons.

**Methods:**

A single blinded, two-arm, randomised controlled trial design was employed. Thirty-eight participants were divided into two intervention arms: Experimental Intervention (EI) group, in which VR and gait-specific physical exercises were administered and Control Intervention (CI) group, in which a placebo virtual gait and the same exercise programme was administered. Functionality, pain, and tone were assessed.

**Results:**

EI group improved in aerobic capacity, functional lower-limb strength, reaction time, and pain, while CI group remained the same. Regarding static balance and muscle tone, no differences were found for either group. Further analysis is needed to asses VR effectiveness for improving gait, stand-up and sit-down performance and velocity.

**Conclusions:**

Virtual running therapy appears to enhance capacities related with voluntary movements (i.e., aerobic capacity, functional lower-limb strength, and reaction time) and reduce pain.

**Supplementary Information:**

The online version contains supplementary material available at 10.1007/s40520-023-02414-x.

## Introduction

Frailty is caused by accumulated age-related deficits in older persons. It has negative consequences on the individual’s overall physical health, muscle tone alteration and weakness, impaired coordination or increased fatigue, which leads to reduced mobility and functionality and thus has a negative impact on quality of life [1]. Altered functionality, in turn, exacerbates all these morbid processes, starting a vicious circle that normally leads to inactivity and a sedentary lifestyle [2].

Therefore, it is important to prescribe therapeutic exercise in this population, in an attempt to break the vicious circle and improve their physical condition [3]. However, training must be sufficiently demanding and long-lasting to achieve the therapeutic objectives and thus a health benefit [4], while this population’s altered physical capabilities may prevent clinicians from prescribing the recommended exercise based on the age-specific exercise capacity threshold. People with frailty may perceive excessive fatigue, which could lead to failure in attaining the objectives of the programme and eventually, abandonment [5].

This handicap makes it difficult to prescribe the required volume and intensity of exercise, as it must be adapted to their poor physical condition. For this reason, there are different therapeutic alternatives to increase the results of exercise, such as those involving the cortical areas that represent movement (i.e., motor and premotor areas) [6, 7], that enhance changes in synaptic structure and, therefore, the plasticity capacity of the nervous system (i.e., neuroplasticity).

It is the case of therapies that use virtual reality to activate mirror neurons, which are defined as a particular type of visuomotor neurons, originally discovered in the F5 area of the premotor cortex of a monkey [8–10]. Nowadays, it is known that these neurons are located both in motor and premotor areas, as well as in other cortical and subcortical areas [11, 12]. This type of neurons show activation, not only when a movement is executed but when a similar motor action is observed and scrutinized [13]. In fact, the main function of mirror neurons is understanding of actions and participate in imitation actions [14]. Thus, when an individual observes an action with a specific goal performed by another individual, the neurons representing this action are activated in the motor and premotor cortex of the observer, transforming the information visually received into knowledge [15–17].

Related therapies are based on the assumption that previously viewing a specific action may improve its subsequent execution since these therapies produce an activation of cortical and subcortical neural networks where the mirror neurons are located, as many studies confirm throw physiological measures [18], enhancing adaptative neuroplasticity mechanisms. In this line, various studies using mirror neuron-based therapy have proved to be effective on several motor functions in populations with stroke, Parkinson's disease, or Alzheimer's disease [19–22].

Further, the effect of mirror neuron therapies was also analysed in other populations with sensitive injury, such as in people with spinal cord injury. Specifically, bodily illusion therapy was effective in reducing neuropathic pain in people with spinal cord injury with promising results [23–25]. Since chronic pain is caused by a disrupted cortical proprioceptive representation, causing an imbalance between the distorted cortical maps of representation of the zones involved in motor control(specifically, the somatosensory cortex is altered) and sensory feedback [26], bodily illusion therapy may restore the balance and reduce chronic pain intensity. To the best of our knowledge, there are no previous studies that assess bodily illusion therapies in frail and pre-frail people. Accordingly, the main objective of this study is to evaluate the effects of a virtual running (VR) treatment combined with specific physical gait exercise (PE) compared to placebo VR treatment combined with PE, on functionality, pain and muscular tone in pre-frail and frail elderly people.

Methods

A single-blinded, two-arm, randomised controlled trial design was employed. This study was approved by the Ethics Committee of the Universitat de València (1675215) and performed in accordance with the latest revision of the Declaration of Helsinki. All participants signed the informed consent to participate in the study. Moreover, this study was registered at ClinicalTrials.gov (NCT05273229).

### Participants

Volunteers aged over 65 years were recruited through different older people’s associations (i.e., *La Nau Gran* and *Asociación Grupo de Mayores de Telefónica*). The inclusion criteria were the following: i. elderly people (> 65 years), ii. pre-frail or frail based on the Fried criteria [27], iii. ability to walk with or without aids, and iv. ability to understand instructions (Mini-Mental State Examination > 23 points). Exclusion criteria were the following: i. traumatic pathology, ii. alterations of the central or peripheral nervous system, iii. alterations of the vestibular system, and iv. concomitant diseases.

### Procedure

In the first visit, eligible volunteers were informed about all study proceedings and an informed consent was signed. In this session, height and weight measurements were obtained. If the volunteer met all the criteria to participate in the study, he/she was assigned to an intervention group with a blinded randomisation method and was scheduled to return another day for the pre-treatment assessment.

Participants were divided into two intervention arms: Experimental Intervention (EI) group, in which VR and gait-specific physical exercises were administered and Control Intervention (CI) group, in which placebo virtual gait and the same exercise programme were administered. The allocation procedure used was the simple randomisation method with the Random Allocation Software conducted by an external assistant who was blinded to the study objectives [28]. Randomisation and disclosure of group assignment occurred after the first evaluation session.

In the second visit, a pre-treatment assessment was conducted by a researcher blinded to group allocation (*T1*). The assessment procedure was repeated by the same researcher at the end of the 8 week treatment (*T*2) and 4 weeks after the end of the intervention (*T*3).

### Interventions

Both interventions (EI and CI) lasted 8 weeks (3 days per week), and the sessions were carried out in groups of 4 people. Researchers recorded any adverse effects in each session.

EI consisted of 10 min of VR and 35 min of PE, while CI included 10 min of placebo VR and 35 min of PE:

- For the VR, the participant was placed in a standing position (with an ad-hoc-designed help system) in front of a mirror (upper body) and a screen (lower body) where a video of legs running on a treadmill was projected (eFigure 1). The projected legs were adapted to each subject according to their height and weight, to achieve the maximum similarity to a real mirror therapy effect ensuring that the participants felt the projected legs as their own.
- For the placebo VR, the set-up and duration were the same as for VR, although videos of landscapes without featuring any type of human or animal movement were projected, since mirror neurons do not report activation in response to the movement of objects [14, 29, 30].
- PE: lower-limb exercises were carried out for 35 min, including coordination, strength, balance and stretch exercises (eTable 1). The Borg Scale was used in each session to measure each person’s perception of their effort and exertion, breathlessness, and fatigue during each physical exercise. This scale was shown high reliability (ICC = 0.85-0-91) [31]. More exercise intensity was added when participants reported less than “regular” effort on this scale. Therefore, heavier loads and unstable surfaces were used for adding higher intensity.

### Functional outcomes

The 2-min Walk Test (2MWT) was used to assess participants’ aerobic capacity. This test recorded the distance covered in 2 min (*m*) and it has excellent reliability (ICC = 0.95) [32].

The Five Times Sit to Stand Test (5xSTS) was used to record the required time (s) to rise from a chair five times. It assessed functional lower-limb strength, and it also has shown excellent reliability (ICC = 0.89) [33]

An inertial sensor embedded in the Android device FallSkip^®^ system (Biomechanical Institute of Valencia, València, Spain) [34] was used according to the protocol previously developed and validated by our group [35]. The device was fixed at the height of L4–L5, approximately coinciding with the centre of gravity. This functional assessment included five phases performed sequentially in a single recording:

Phase 1. Standing still with arms alongside the body for 30 s.

Phase 2. Walking straight ahead as fast and as safely as possible towards a chair 3 m away at the sound of an acoustic signal.

Phase 3. Turning around and sitting down in a chair.

Phase 4. Standing up from the chair.

Phase 5. Walking back as fast and as safely as possible to the starting point.

Three variables were calculated for the static postural control phase: i. Medial–lateral (ML) displacement of the centre of mass (in mm); ii. Anterior–posterior (AP) displacement of the centre of mass (in mm); and iii. Displacement area of the centre of mass (in mm^2^). With respect to gait analysis in phase 2, two variables were measured: i. Vertical (V) range of the centre of mass (in mm); ii. ML range of the centre of mass (in mm) [35].

Likewise, turning around and sitting down and standing up from the chair were also monitored and three variables were calculated: i. Standing-to-sitting time (in s); ii. Standing-up power (in W); and iii. Sitting-to-standing time (in s). Finally, two time-related variables were calculated: i. Total time (s); ii. Reaction time from a sound stimulus (s) [35].

### Tone outcomes

The myometer MyotonPRO^®^ (Myoton AS, Tallinn, Estonia) was used to explore the impact on the dominant lower-limb muscle tone. The reliability of MyotonPRO^®^ has been demonstrated for the evaluation of muscle properties [36–38] and the measurement procedures were performed as described previously [39, 40]. In this study, lower-limb muscles (i.e., tibialis anterior, rectus femoris of the quadriceps, gluteus major, biceps femoris of the hamstrings, and gastrocnemius lateralis muscles) were assessed.

First of all, a line was drawn between the two anatomical landmarks of the given muscle, following the manufacturer’s recommendations. Then, the assessment point (centre of the muscle belly) was identified on that line, at the largest cross-section of the muscle belly, and those points were marked with a pen. The outcomes used were Natural Oscillation Frequency (Hz), characterising tone or tension, and Dynamic Stiffness (N) that measures tissue resistance to external and internal deformation at rest.

### Pain outcomes

10 cm Visual Analogue Scale (VAS), 0 being no pain and 10 being the worst pain, was used to explore impact of the intervention on maximum and mean pain intensity. The VAS is an established, validated, and reliable measure of pain intensity (0.90) [41, 42].

### Statistical analysis

Statistical data analysis was conducted using SPSS v28 (Inc., Chicago, IL, USA). Classical statistical methods were used to obtain the mean as a central measure of trend and the standard deviation (SD) as a measure of dispersion. A split-plot ANOVA with the within-subject factor “Time” (i.e., *T*1, *T*2 and *T*3) and a between-subject factor “Group” (i.e., EI and CI) was used to search for differences in outcome measures cited above. Normality was checked using Shapiro–Wilk, homoscedasticity, using Levene’s test and sphericity using Mauchly’s test. Type I error was set at 5% (*p* ≤ 0.05).

## Results

**Fig. 1:**
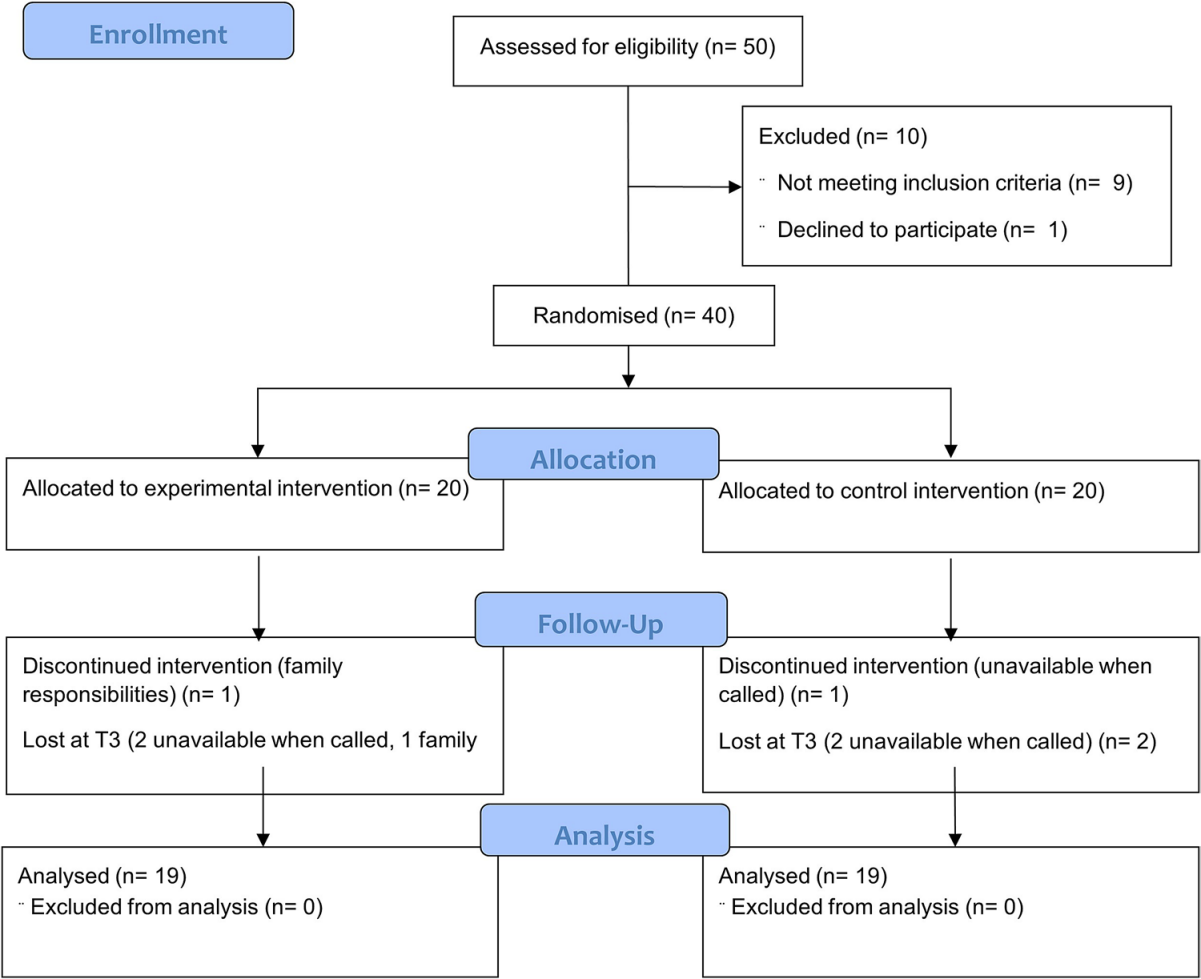
CONSORT flow diagram

The study included 38 pre-frail and frail elderly people, 19 allocated to EI group and 19 allocated to CI group (Fig. 1). Demographic data are listed in Table 1 and no significant differences were found for age, height, weight and sex between groups.

**Table 1:** Demographic data

	EI		CI	
	Mean	SD	Mean	SD
Age (years)	71.68	4.40	72.26	3.33
Height (cm)	159.50	5.94	151.14	37.72
Weight (kg)	73.44	16.93	72.58	16.51

	Men	Women	Men	Women
Sex (*n*)	2	17	3	16

*CI* control intervention group *EI* experimental intervention group

### Functional outcomes

Table 2 shows results related to functional outcomes. People from the EI group significantly improved the distance covered in 2 min (2MWT), decreased time to perform 5xSTS and decreased reaction time between *T1* and *T*2, maintaining this improvement until *T3*. By contrast, people from CI group did not significantly improve any of the assessed functional outcomes (Table 2).

**Table 2:** Functional outcome results

	EI group			CI group		
	*T*1	*T*2	*T*3	*T*1	*T*2	*T*3
2MWT						
2MWT (*m*)	147.74(29.24)ab	157.86 (28.42)	160.07 (21.67)	147.23 (24.46)	151.61 (19.83)	149.98 (20.06)
5xSTS						
5xSTS (seconds)	14.75 (5.28)ab	11.75(2.92)	10.79 (2.48)	14.52 (4.11)	12.42 (2.26)	12.39 (4.03)
FallSkip						
Balance (ML displacement, mm)	6.91 (2.92)	8.25 (4.61)	7.01 (5.11)	6.59 (2.76)	6.34 (3.08)	7.28 (2.75)
Balance (AP displacement, mm)	18.55 (8.41)	18.66 (6.91)	20.55 (11.38)	16.49 (4.58)	19.51 (6.21)	19.38 (4.21)
Balance (Area, mm^2^)	381.79 (257.19)	468.25 (339.82)	492.04 (570.73)	308.93 (145.66)	368.057 (226.11)	417.05 (222.25)
Gait (V Range, mm)	30.53 (6.36)	33.13 (9.74)	32.26 (8.34)	27.59 (4.58)	28.98 (5.18)	28.39 (4.78)
Gait (ML Range, mm)	47.53 (14.47)	51.68 (17.83)	47.51 (13.13)	46.39 (15.06)	43.11 (12.99)	44.46 (12.11)
Standing up Power (*w*)	202.15 (64.37)	217.84 (80.51)	185.64 (66.17)	213.50 (70.14)	183.43 (54.57)	191.18 (102.95)
Total time (*s*)	11.91 (2.14)	11.14 (1.42)	11.22 (1.18)	12.12 (1.39)	12.11 (1.52)	12.31 (1.96)
Standing-to-sitting time (s)	2.91 (0.85)	3.02 (0.87)	2.67 (0.41)	3.02 (0.69)	3.29 (0.77)	3.17 (0.88)
Sitting-to-standing time (*s*)	1.58 (0.59)	1.45 (0.36)	1.67 (0.54)	1.43 (0.37)	1.63 (0.39)	1.75 (0.79)
Time to react (s)	1.09 (0.43)ab	0.80 (0.32)	0.70 (0.25)	0.73 (0.29)	0.89 (0.29)	0.80 (0.23)

*EI* experimental intervention*, CI* Control intervention, *2MWT* two-minute walk test, *5xSTS* five times sit to stand, *SPPB* short physical performance battery, *T1* pre-treatment assessment, *T2*: post-treatment assessment; *T3*: 1 month follow-up

^a^Significant differences between *T*1 and *T*2 (*p* < 0.05)

^b^Significant differences between *T*1 and *T*3 (*p* < 0.05)

### Tone outcomes

Regarding lower-limb tone outcomes, no significant differences were found between assessments in either of the groups (Table 3).

**Table 3:** Tone results in each of the assessments performed

		EI group			CI group		
		T1	T2	T3	T1	T2	T3
Tibialis anterior	Frequency (Hz)	20.17 (2.52)	20.33 (2.66)	19.83 (2.76)	20.07 (2.35)	20.11 (2.35)	20.49 (2.46)
	Stiffness (N/m)	412.37 (74.24)	420.05 (78.91)	399.14 (105.01)	409.74 (76.68)	412.63 (69.67)	414.22 (69.47)
Rectus femoris of the quadriceps	Frequency (Hz)	12.81 (1.77)	12.06 (1.63)	13.27 (1.93)	12.08 (1.12)	12.51 (1.63)	12.28 (1.33)
	Stiffness (N/m)	261.37 (31.65)	259.58 (32.26)	274.21 (36.60)	256.47 (31.64)	266.63 (32.26)	259.83 (31.10)
Gluteus major	Frequency (Hz)	11.43 (1.65)	12.14 (2.53)	11.67 (1.51)	11.92 (2.65)	13.25 (5.19)	13.76 (6.03)
	Stiffness (N/m)	230.21 (21.67)	240.74 (24.89)	218.75 (63.12)	218.90 (59.01)	244.42 (32.63)	247.50 (35.12)
Biceps femoris of the hamstrings	Frequency (Hz)	15.48 (1.65)	16.39 (3.15)	15.86 (1.61)	15.19 (1.76)	15.06 (2.14)	15.21 (1.52)
	Stiffness (N/m)	283.00 (36.99)	293.16 (36.82)	288.64 (34.13)	284.37 (3.15)	281.00 (35.62)	275.33 (36.21)
Gastrocnemius lateralis	Frequency (Hz)	16.18 (2.66)	15.52 (2.86)	15.78 (1.82)	15.27 (2.15)	15.50 (1.90)	15.59 (1.89)
	Stiffness (N/m)	297.42 (5.24)	292.32 (52.22)	295.07 (34.81)	294.00 (39.70)	298.84 (41.09)	292.68 (39.39)

Data are expressed as mean (SD). *

### Pain outcomes

**Fig. 2:**
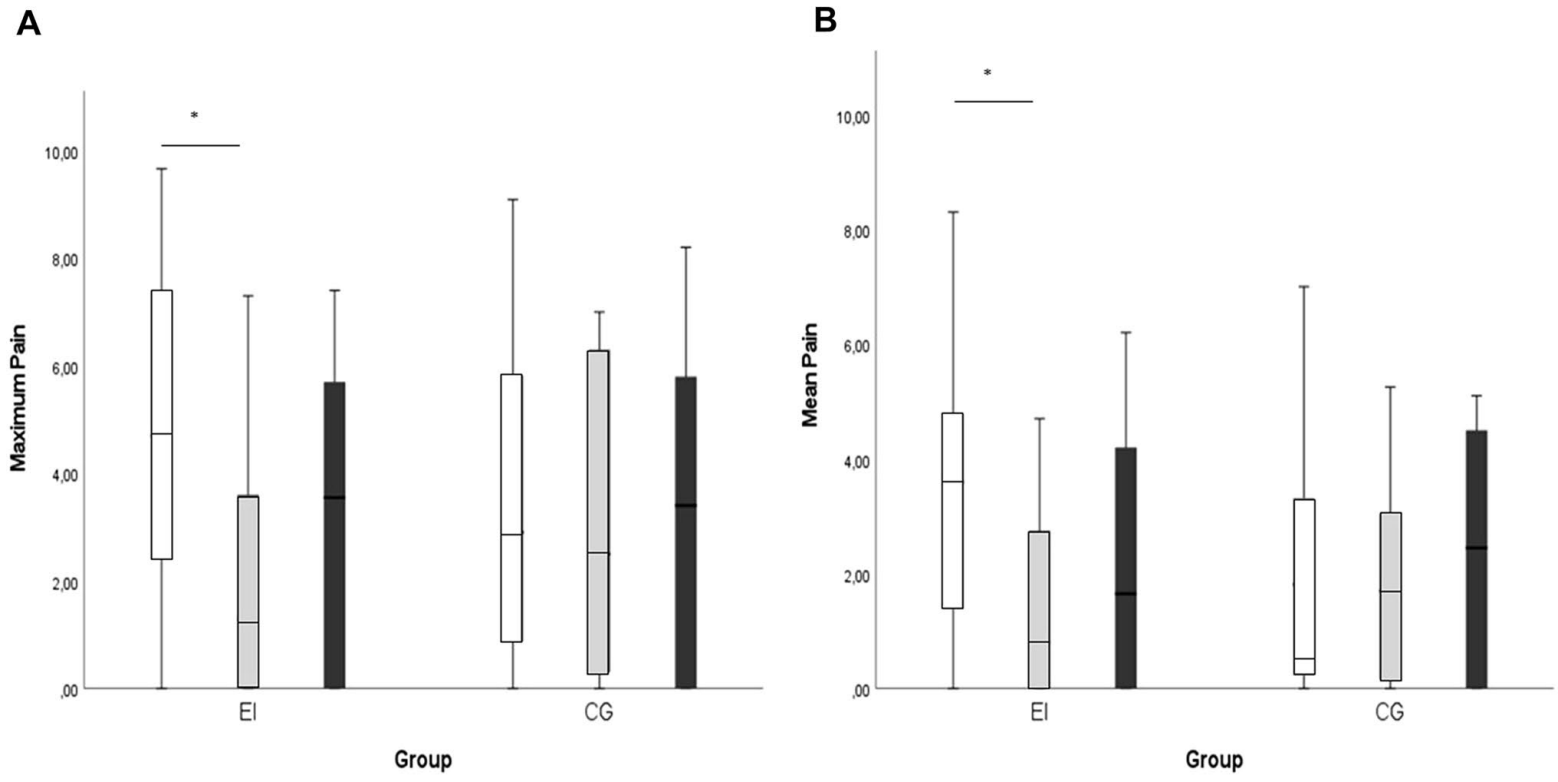
Previous week reported maximum (**A**) and mean (**B**) pain. White bars represent the pre-treatment assessment (*T*1), the grey ones the post-treatment assessment (*T*2) and the black bars, the 1 month follow-up assessment (*T*3). *Significant differences. *CI* control intervention, *EI* experimental intervention

Figure 2 shows pain results. EI group shows a significant pain decrease at *T*2 compared to *T*1 in both pain domains assessed, while pain levels in CI group remained at the same level during all assessments.

## Discussion

This study aimed to evaluate the effectiveness of a VR treatment combined with specific PE compared to placebo VR treatment combined with PE in improving functionality, tone and stiffness, and pain in pre-frail and frail elderly people. It was based on the hypothesis that VR therapy could potentiate the effects of conventional exercise in a pre-frail and frail population, even when failing to reach the minimum exercise capacity threshold as per WHO recommendations because of their fatigue (assessed with the Borg Rating of Perceived Exertion). Therefore, this is the first study to provide the scientific community with an understanding on how the mechanisms of neuroplasticity and specifically mirror neurons can influence the effect of the physical training on functionality in frail and pre-frail elderly people.

For this reason, functionality, and muscle tone and stiffness were assessed since the expected improvement in physical condition could imply an improvement in certain physical capacities and muscle tone. Specifically, this study assessed aerobic capacity with 2MWT; functional lower-limb strength with 5XSTS test; static balance, gait performance and velocity, standing-up power, sitting-to-standing and standing-to-sitting times, and reaction time with inertial sensors (FallSkip tool); and tone and stiffness with Myoton. All these outcomes become important in pre-frail and frail elderly people during activities of daily living [43].

In general, people from the CI group failed to achieve significant functional improvements. This lack of significant improvements using only a PE programme could be due to the fact that the latter was adapted to their level of fatigue, taking into consideration their frailty state, as explained at the methods section.

Nevertheless, when mirror neuron-based therapy was applied in the EI group, an overall improvement in voluntary movement-related capacities was achieved (i.e., aerobic capacity, functional lower-limb strength, and reaction time), with the exception of gait performance and velocity, standing-up power, and sitting-to-standing and standing-to-sitting times. Further, capacities associated with involuntary movements (i.e., static balance, and muscle tone and stiffness) remained similar between assessments in both groups.

As reported in previous studies, mirror neuron system-based therapies could help to reduce the deterioration of motor capabilities by evoking a greater neural activation of the cortical-subcortical network that supervises motor control [44, 45]. The repetitive activation of the mirror neuron network could produce more efficient motor patterns [46]. This was noted in EI group, where optimised motor patterns during voluntary movements resulted in a more efficient movement as shown by the improvement of the aerobic capacity (i.e., 2MWT), functional lower-limb strength (i.e., 5xSTS test) and reaction velocity (i.e., Time to React with FallSkip tool), even maintaining the improvement one month after finishing the intervention. In fact, mirror neurons are the basis of the perceptual-motor transformation mechanism, which allows subjects to transform visual information into motor commands [30]. Therefore, electrophysiological studies have provided extensive evidence for corticospinal reorganization after intervention with a program of stimulation of the mirror neurons [47]. This cortical reorganization achieved both by the virtual gait and the physical exercise program [48–50], would justify that in the EI there will be an improvement maintained up to 1 month after the end of the intervention. Despite the fact that people in EI group showed significant improvements on 2MWT and 5xSTS test, no differences between assessments were found on specific gait performance (i.e., vertical and medial–lateral range during gait and gait velocity), standing-up power and duration, and standing-to-sitting time. This lack of improvement in gait performance and velocity would likely be due to the short distance used for gait assessment (i.e., 3 m). Indeed, gait performed in a clinical context cannot reveal its natural efficiency strategies because of the reduced space available for the assessment [51, 52]. In this way, more studies are needed to assess gait in a more common context for the volunteers. On the other hand, sitting-to-standing and standing-to-sitting outcomes assessed with FallSkip relied only on one repetition, while 5 repetitions were conducted for 5xSTS test, obtaining a more accurate measure.

Regarding involuntary movements, specifically those registered during the static balance phase, no significant differences were found between assessments in either group (i.e., ML displacement, AP displacement, displacement area). This was probably because balancing requires rapid and accurate involuntary movements responding to visual, vestibular, and proprioceptive information [53]. Nevertheless, considering that the mirror neuron system acts in voluntary movement facilitation, different dynamic balance and body centre of pressure control activities should be tested in future research, to be able to test the effect of the mirror neuron system activation on dynamic postural control.

With regard to tone (i.e. natural oscillation frequency) and stiffness, no significant differences were found after the interventions. We decided to include the analysis of the impact of physical intervention on muscle tone, since physical exercise has been considered important for muscle elasticity [54], and we wanted to assess whether VR could affect these results. However, no significant improvement was obtained in either group and VR showed no impact on tone behaviour.

Different peripheral and central mechanisms are associated with tone changes [55], including cortex, basal ganglia, cerebellum, brainstem reticular system, spinal cord and muscle spindle [56]. Further, tone also has a viscoelastic component which is independent of neural activity, including sarcomere actin-myosin cross-bridges, the viscosity, elasticity, and extensibility of the contractile filaments, filamentous connection of the non-contractile proteins of sarcomeres, osmotic pressure of the cells, and also pressure on the surrounding connective tissues [56]. In this way, the mirror neuron system has no potential to impact on this viscoelastic component. The influence of more demanding PE could be tested to explore whether the combination of VR and more demanding PE may affect tone properties.

Regarding pain outcome, previous studies have shown promising results for the treatment of pain using movement imagery [57, 58], mirror therapy [59, 60] or ‘virtual’ mirror therapy [23–25]. In this study, both pain domains assessed improved in EI group between T1 and T2, while pain levels in CI group remained the same throughout the study. Pain relief involves an improved proprioceptive representation, disrupting the imbalance between motor cortical representation and distorted sensory feedback produced by age-related injuries that could cause chronic unspecific pain [26]. Therefore, mirror neuron system activation could produce a normalisation of cortical somatosensory representation maps and, in addition, contribute to modulate cortical and spinal excitability [60–64]. However, these changes are not maintained at the T3 assessment, so longer studies are needed to analyse long-term therapy effects on non-specific pain.

This study has some limitations. Firstly, because of the nature of therapy, it was difficult to totally blind therapists and participants. Secondly, the sample has a greater representation of women than men, therefore, this must be taken into account for the generalisation of results. Finally, purposive sampling for the recruitment of the volunteers was used instead of simple randomisation.

## Conclusion

Based on all the foregoing, this study provides evidence that neuron system activation through VR therapy can improve voluntary movement-related capacities (i.e., aerobic capacity, functional lower-limb strength, and reaction time) and pain, while VR therapy has no effect on capacities and characteristics associated with involuntary movement (i.e., static balance and muscle tone and stiffness) in frail and pre-frail people.

## Supplementary Information

Below is the link to the electronic supplementary material.

Supplementary file 1 (JPG 389 KB) Set-up protocol.

Supplementary file 2 (DOCX 399 KB)

## Data Availability

The datasets generated during and/or analyzed during the current study are available from the corresponding author on reasonable request.
