# Supplementary figures and images for: Effect of virtual running with exercise on functionality in pre-frail and frail elderly people: randomized clinical trial
Source: Aging Clin Exp Res. 2023 May 15;35(7):1459–67. doi: 10.1007/s40520-023-02414-x (PMC10284997; doi:10.1007/s40520-023-02414-x)

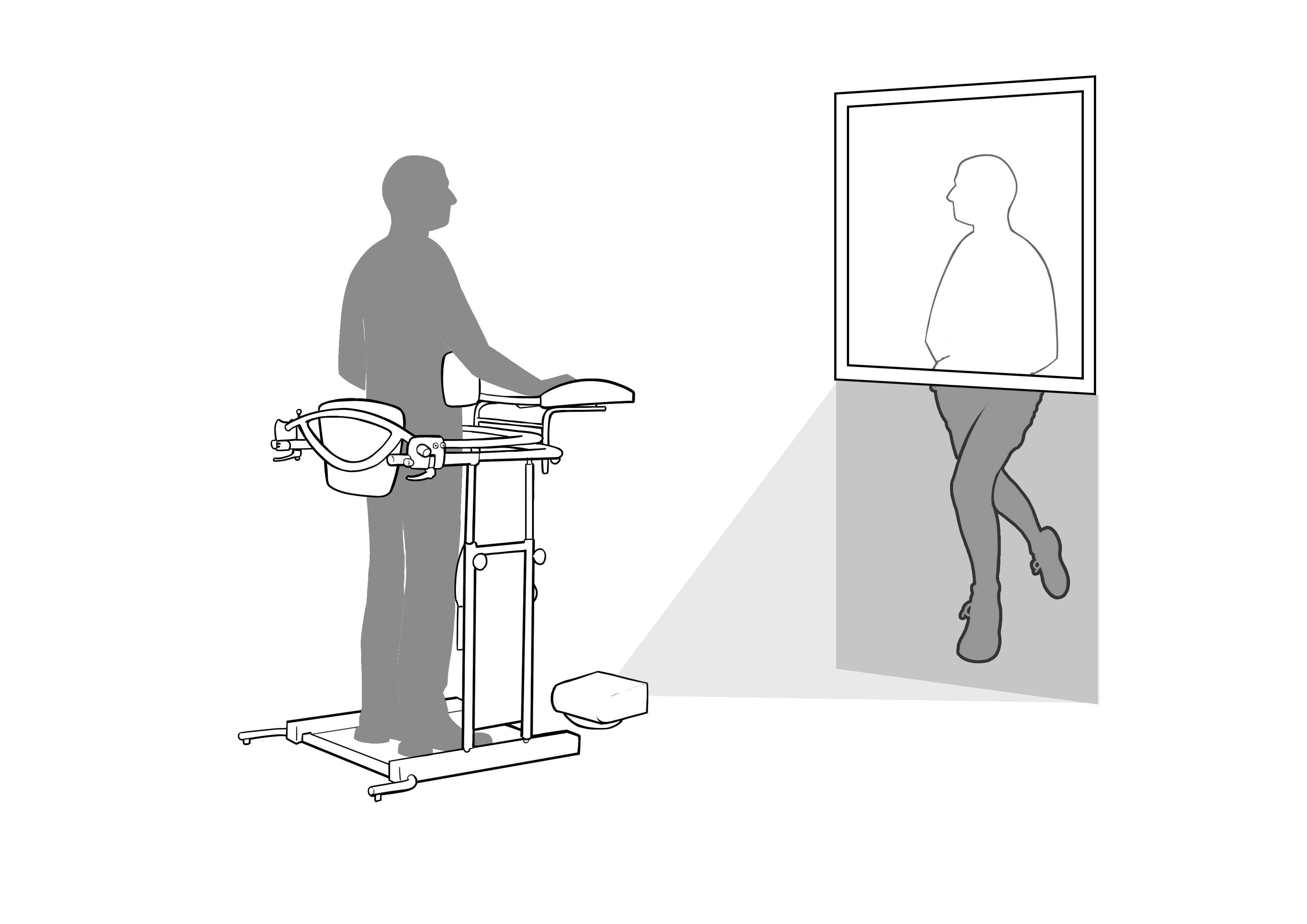

Supplement: Supplementary file 1 — (JPG 389 KB) Set-up protocol. [file 40520_2023_2414_MOESM1_ESM.jpg]
