# Supplementary material for: Effect of virtual running with exercise on functionality in pre-frail and frail elderly people: randomized clinical trial
Source: Aging Clin Exp Res. 2023 May 15;35(7):1459–67. doi: 10.1007/s40520-023-02414-x (PMC10284997; doi:10.1007/s40520-023-02414-x)
Supplement: Supplementary file 2 — (DOCX 399 KB) [file 40520_2023_2414_MOESM2_ESM.docx]

|  | Exercise | Figure | Series, repetitions | Advanced form |
| --- | --- | --- | --- | --- |
| Coordination exercises | Normal walking with obstacles | 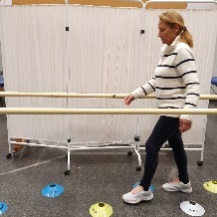 | 3, 3 | Higher speed |
|  | Lateral walking with obstacles | 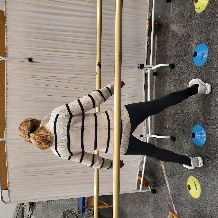 | 3, 3 | Higher speed |
|  | Backward walking | 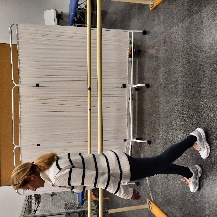 | 3, 3 | Higher speed |
| Strength exercises | Squat | 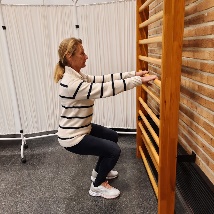 | 3, 10 | Adding a Bosu |
|  | Ankle flexion-extension with body weight | 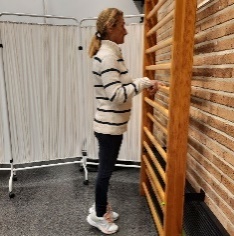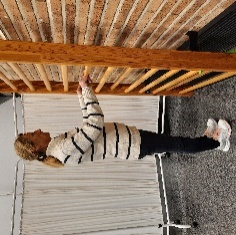 | 3, 10 | Adding weight |
|  | Hip abduction | 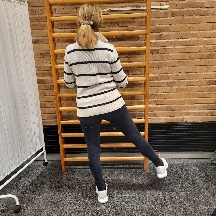 | 3, 10 | Adding weight |
|  | Knee flexion in prone position with weights | 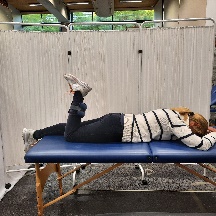 | 3, 10 | Adding weight |
| Balance exercise | Monopodal balance | 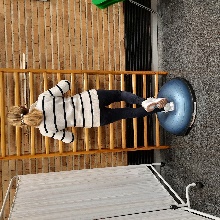 | 3, 1min | With a Bosu |
| Stretching exercises | Lower-limb passive stretching | 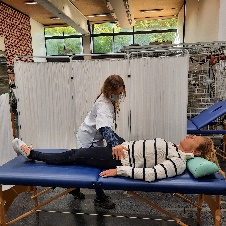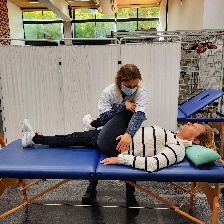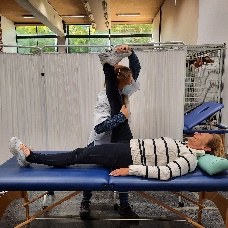 | 1, 30 s | NA |

All the exercises were performed guaranteeing a regular effort perception corresponding with a score of 12-13 on the Borg scale.
